# In-situ observation of the initiation of plasticity by nucleation of prismatic dislocation loops

**DOI:** 10.1038/s41467-020-15775-y

**Published:** 2020-05-12

**Authors:** Subin Lee, Aviral Vaid, Jiseong Im, Bongsoo Kim, Arun Prakash, Julien Guénolé, Daniel Kiener, Erik Bitzek, Sang Ho Oh

**Affiliations:** 1 Department of Materials Science and Engineering, Pohang University of Science and Technology (POSTECH), Pohang, 37673 Republic of Korea; 2 Center for Integrated Nanostructure Physics, Institute for Basic Science (IBS), Suwon, 16419 Republic of Korea; 3 Department of Materials Science and Engineering, Institute I: General Materials Properties, Friedrich-Alexander-Universität Erlangen-Nürnberg, 91058 Erlangen, Germany; 4 Department of Chemistry, KAIST, Daejeon, 34141 Korea; 5 Department of Materials Science, Montanuniversität Leoben, Jahnstraße 12, 8700 Leoben, Austria; 6 Department of Energy Science, Sungkyunkwan University (SKKU), Suwon, 16419 Republic of Korea

**Keywords:** Nanowires, Structural properties, Metals and alloys, Transmission electron microscopy

## Abstract

The elastic-to-plastic transition during the deformation of a dislocation-free nanoscale volume is accompanied by displacement bursts associated with dislocation nucleation. The dislocations that nucleate during the so-called “pop-in” burst take the form of prismatic dislocation loops (PDLs) and exhibit characteristic burst-like emission and plastic recovery. Here, we report the in-situ transmission electron microscopy (TEM) observation of the initial plasticity ensued by burst-like emission of PDLs on nanoindentation of dislocation-free Au nanowires. The in-situ TEM nanoindentation showed that the nucleation and subsequent cross slip of shear loop(s) are the rate-limiting steps. As the indentation size increases, the cross slip of shear loop becomes favored, resulting in a transition from PDLs to open half-loops to helical dislocations. In the present case of nanoindentation of dislocation-free volumes, the PDLs glide out of the indentation stress field while spreading the plastic zone, as opposed to the underlying assumption of the Nix-Gao model.

## Abstract (web summary)

Prismatic dislocation loops (PDLs) form during the elastic-to-plastic transition of a dislocation-free volume under nanoindentation. Here the authors observe the initial plasticity and burst-like emission of PDLs in Au nanowires by in-situ transmission electron microscopy, elucidating fundamental aspects of the formation process.

## Introduction

As the characteristic length of deformation volume or testing sample decreases down to the nanometer scale, dislocation nucleation plays a dominant role in the yield and subsequent plastic deformation behavior, as opposed to the glide and multiplication of pre-existing dislocations in bulk^1–6^. The onset of plasticity by dislocation nucleation can be assessed experimentally by nanoindentation of a dislocation-free volume^7–9^, where the yield point is usually followed by a sudden displacement burst called pop-in^7,10,11^. Some fundamental questions related to this incipient plasticity and subsequent pop-in are: whether the first dislocation nucleates prior to or just at the pop-in^12,13^, and whether it nucleates via a homogeneous^3,4,14^ or heterogeneous process^15–19^.

The collective emission of prismatic dislocation loops (PDLs) has been attributed as the underlying dislocation process of pop-in bursts^20–25^. These PDLs can extend along specific crystallographic directions far beyond the plastic zone underneath the indent, where dislocation accumulations are commonly expected^21,26^. Since nanoindentation introduces strain gradients, PDLs represent a natural realization of geometrically necessary dislocations (GNDs) that accommodate the local shape change imposed by the indenter^1,27^. As such, PDLs have played a central role in the models developed to account for the indentation size effect or strain gradient-based strengthening^1,28^. Therefore, the mechanisms of formation, glide, and interaction of PDLs are of prime importance in understanding the yield and pop-in phenomena and the mechanical properties at nanometer scale^6^.

So far, the formation mechanism of PDLs has been studied mainly by molecular dynamics (MD) simulations^4,24,25,29^ due to the difficulties of direct observation of the formation process and the limited information gained by post-mortem transmission electron microscopy (TEM) analysis of PDLs. Different mechanisms have been suggested, which involve either cross slip of a single dislocation^4,30^ or the nucleation of multiple dislocations and reactions between them^25,31^. However, an information gap nevertheless exists between the formation mechanisms studied by MD simulations and those inferred from the post-mortem TEM observation of dislocation structures. In-situ TEM nanoindentation, which can directly visualize the evolution of dislocation structures from the incipient plasticity to subsequent PDL emission, can greatly improve our understanding.

Here, we present in-situ TEM observations of the formation of PDLs during the pop-in burst in nanoindentation, which was enabled by establishing an asperity contact to the sharp tip of defect-free Au [110] nanowires in TEM. These in-situ TEM observations showed that the formation of PDLs proceeds by the nucleation of shear loop(s) within the plastic zone. This event is the first rate-limiting step, requiring the accumulation of elastic strain energy to overcome the related energy barrier. Following the nucleation, a cross slip of the shear loop to differently oriented slip planes is the second rate-limiting step to the formation of PDL, which competes with the nucleation of another shear loop on the corresponding slip plane. As detailed later, the cross slip process is favored when the line length of the screw components increases, which is responsible for the transition from PDLs to helical dislocations at later stages of nanoindentation.

## Results

### Asperity contact and PDL formation in TEM nanoindentation

**Fig. 1:**
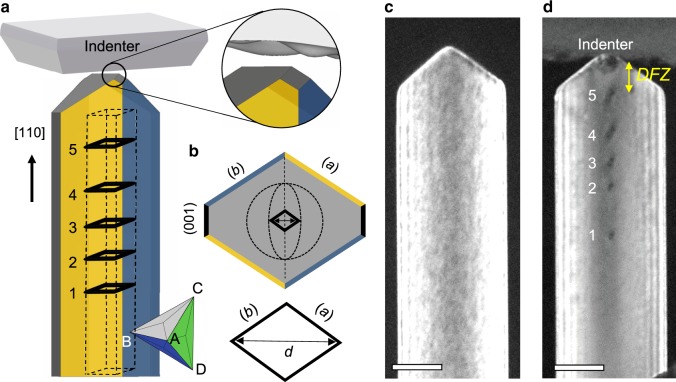
Emission of PDLs during nanoindentation. **a** Schematic illustration showing an asperity contact at the sharp tip of the nanowire and PDLs emitted from the contact. The nanowire has a truncated rhombic cross-sectional shape bound by parallel $(\bar{1}11)$, $(1\bar{1}1)$ (or (*b*), (*a*)) and (001) planes. The tip of the nanowire is facetted by inclined (*c*) and (*d*) planes into a wedge shape. An array of PDLs lying on a rhombic glide prism is represented by black lines. An asperity contact formed at the nanowire tip due to the roughness of the truncated diamond indenter is represented schematically. **b** Top view of the schematic model of nanowire with hypothetical contact line and PDL. The elliptical contact line made by a hypothetical round-shaped asperity on the indenter is outlined by dotted lines. The size of PDL (*d*) is defined as the long diagonal of the rhombus. **c** TEM dark-field (DF) image of a dislocation-free single crystal Au [110] nanowire before indentation. The viewing direction is [$\bar{1}10$], along which the wedge-shaped sharp tip of the nanowire is visible. Au nanowires with diameters ranging between 130 and 430 nm were used without thinning for TEM, as they are transparent to the 200 kV electron beam (Supplementary Fig. 3). **d** TEM DF image after in-situ nanoindentation. An array of PDLs formed during the nanoindentation is visible, numbered in the order of formation. The dislocation-free zone (DFZ) is defined as the distance between the last PDL and the contact point. The scale bars are 100 nm.

We used dislocation-free Au [110] nanowires grown vertically on a SrTiO_3_ substrate^32^ and prepared TEM samples suitable for in-situ nanoindentation of individual nanowires^33^. The sidewalls of the nanowires are facetted mainly by {111} planes, with a small portion of {001} planes at their intersections, into a truncated rhombic cross-section (see Fig. 1a). The top end of the nanowires, that are indented, have a sharp wedge shape bound by two inclined {111} facets (Supplementary Figs. 1–3). When a flat diamond indenter is pressed onto this sharp nanowire tip, an asperity contact is always established due to the unavoidable roughness of the indenter surface. Therefore, the initial deformation is confined into the small volume under the contact (Fig. 1a), and the developed stress field under the contact is comparable to the typical indentation stress field (Supplementary Figs. 4 and 5).

In all our in-situ TEM nanoindentation experiments the initial plastic deformation proceeded by successive emission of PDLs (Supplementary Movie 1). The Burgers vector of the PDLs, **b**, is determined as $a/2[\bar{1}\bar{1}0]$ by applying **g** ⋅ **b** invisibility criterion, where **g** is the reciprocal lattice vector for the respective *hkl* reflection (Supplementary Fig. 6). This Burgers vector corresponds to *CD* in the Thompson tetrahedron notation used throughout this work^34,35^ (Fig. 1a). PDLs glide along a rhombic glide prism enclosed by two sets of parallel (*a*) and (*b*) slip planes, which extends along the $[\bar{1}\bar{1}0]$ indentation axis (Fig. 1a, b). After emission, the PDLs are driven by the indentation stress field until they reach a stable position. As a consequence, a dislocation-free zone (DFZ) remains between asperity contact and emitted PDL (Fig. 1c, d). During successive emission events, the previously formed PDLs are repelled by the stress fields of the subsequent PDL and are forced to glide further away from the contact, extending the PDL array. This stress balance is the reason that the coaxially aligned PDL array extends in regular intervals along the $[\bar{1}\bar{1}0]$ wire axis.

### Nucleation stress of PDL

**Fig. 2:**
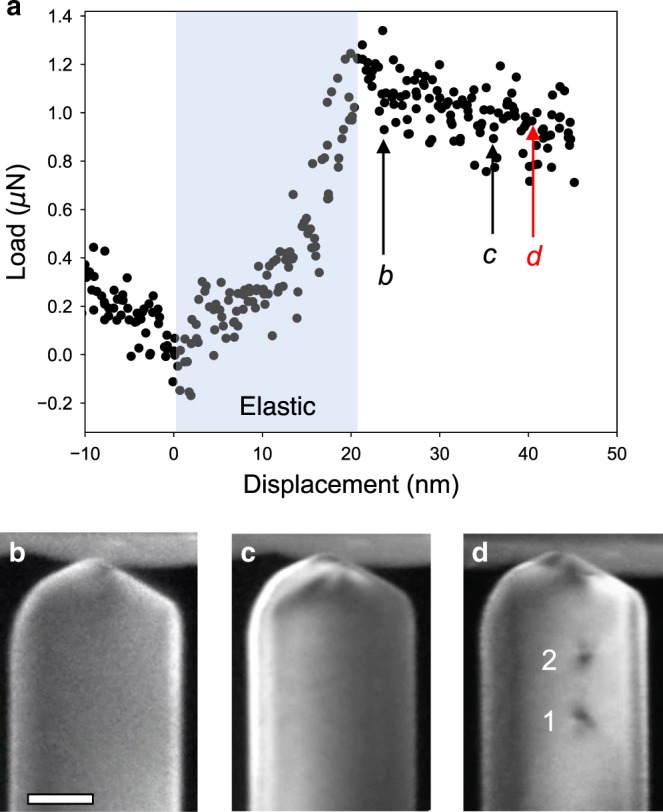
Load-displacement curve and correlated in-situ TEM images. **a** Load-displacement curve and in-situ TEM DF images at the displacement of: **b** the onset of plasticity (the deviation from the elastic loading); **c** before the formation of a PDL; and **d** after the formation of PDLs (Supplementary Movie 2). The PDLs are numbered in the order of formation. We note that fitting the elastic loading regime according to the Hertzian contact model to determine the contact radius is inadequate since the present indentation is not made on a semi-infinite flat surface. Instead, the contact radius at the moment of PDL formation was determined by measuring the size of PDL, which is ~12 nm. The scale bar is 50 nm.

The in-situ TEM nanoindentation of Au nanowires with simultaneous load-displacement measurement revealed that the plasticity of nanowires, as defined by the deviation from the elastic load-displacement curve (point b in Fig.  2a), is not caused by the formation or glide of the first PDL (Supplementary Movie 2). Rather, its formation takes place after yielding and requires substantial elastoplastic loading (point d in Fig. 2a). The mean contact pressure, *p*_0_, at the formation of the first PDL is defined as *p*_0_ = *P*/*πa*^2^, where, *P* is the normal load and *a* is the contact radius^36^. The contact radius can be assessed by measuring the size of PDL on TEM images, as our MD simulation shows the loop size (*d*, refer to the definition in Fig. 1a) increases linearly with the contact radius (*a*) (Supplementary Fig. 7). Using the normal load *P* = 0.8 μN and *a* = 12 nm, the *p*_0_ is calculated to be 1.8 GPa, which is comparable to the ideal strength of Au (0.9–4.3 GPa)^37,38^. We note that the stress for the nucleation of embryonic dislocations initiating the plasticity, considering the higher load and the smaller contact area at the yield point (point b in Fig. 2a), must be higher than that of the first PDL. However, the nucleation of embryonic dislocations at the onset of plasticity and the subsequent formation of PDLs have been often conjectured rather than observed experimentally as these processes happen extremely rapidly and under high stress concentrations^4^.

### Nucleation of stable shear loops as source for PDLs

**Fig. 3:**
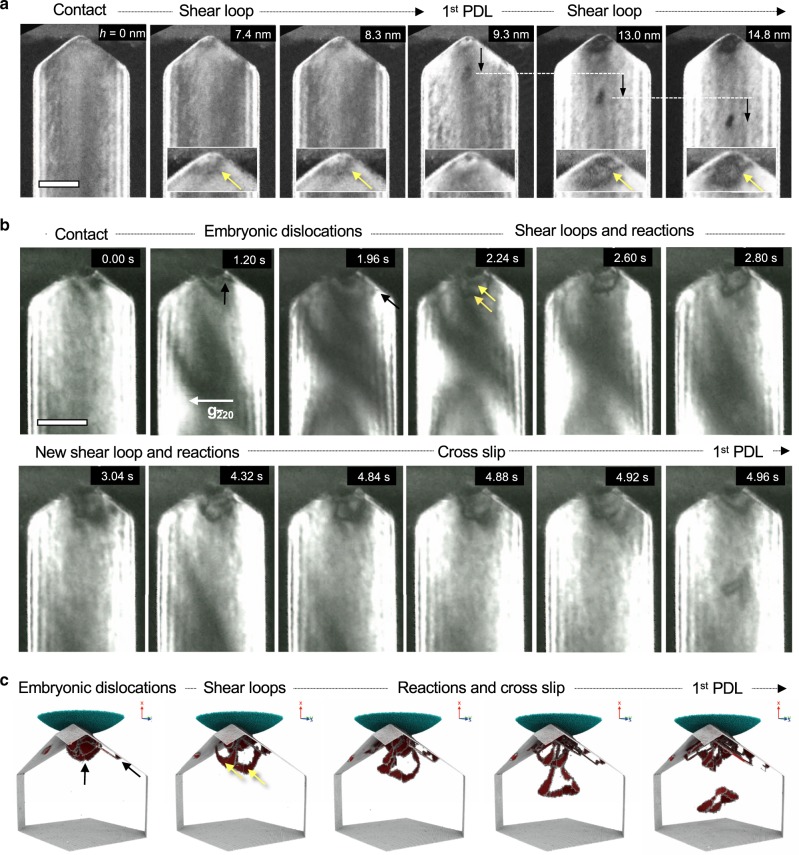
Formation process of PDLs observed by in-situ TEM nanoindentation. **a** A series of TEM DF images showing the formation of a PDL under a small contact area made to a 130 nm thick nanowire (Supplementary Movie 3). The first PDL is punched out quickly upon contact formation. Detailed image analysis reveals that: a shear loop (yellow arrow in magnified inset image at the indentation depth *h* = 7.4 nm) nucleates, expands under the indentation stress field, punches out a PDL (black arrow, *h* = 9.3 nm) and retracts. During subsequent loading (from *h* = 9.3 nm to 14.8 nm) the formation of new shear loop with a larger size than the previous one (yellow arrow, *h* = 13.0 nm) requires the accumulation of more elastic energy, delaying the formation of subsequent PDLs. **b** A series of TEM DF images showing the formation of a PDL under a large contact area made to a 200 nm thick nanowire (Supplementary Movie 4). In the beginning, embryonic dislocations nucleate in the indented region and glide not only along the parallel (*a*) and (*b*), but also the inclined (*c*) and (*d*) slip planes (black arrows). The embryonic dislocations nucleated along the inclined (*c*) and (*d*) slip planes take the form of a half prismatic dislocation loop and glide out of the nanowire. The embryonic dislocations nucleated along the (*a*) and (*b*) planes, which are parallel to the indentation direction, are stored in the plastic zone. Among them two embryonic dislocations are triggered to evolve into stable shear loops (yellow arrows) along the (*a*) or (*b*) planes, which interact to form an extended dislocation and act as source of the PDL. During further loading, the extended dislocation exhibits a complex line shape. Another shear loop grows out on a parallel (*a*) plane and interacts with the existing dislocation structure (3.04 s and forward). The diffraction contrast changes most abruptly when the shear loops form, e.g., at 1.96 s and 3.04 s, indicating a large stress/strain relaxation. After cross slip, the dislocation develops a lasso-like line shape (4.92 s) before pinching-off a PDL (4.96 s). The shear loop shrinks back to the pinning points while the detached PDL glides away, driven by the indentation stress field. **c** A series of MD snapshot images corresponding to the major stages of the formation of the first PDL. Scale bars: **a** 50 nm, **b** 100 nm.

The formation of PDLs as well as the nucleation of embryonic dislocations was captured by implementing elaborate in-situ TEM nanoindentation experiments and analyzed in detail (Fig. 3). During the elastic loading, the TEM diffraction contrast changes gradually in response to the evolution of the indentation strain field. Thereafter, the diffraction contrast changes abruptly with the onset of plasticity, as the strain field is altered by the nucleation of dislocations (Supplementary Movies 1–4). Afterward, a clearly visible PDL is emitted from the indentation region.

We observed two different formation processes of PDLs depending on the actual contact area. In the case of a small contact area (contact radius <5 nm, Fig. 3a) as observed for a 130 nm thick Au nanowire, a PDL was quickly punched out after contact i.e., with fewer prior dislocation activities (Supplementary Movie 3); only one shear loop was seen to nucleate from the contact and dragged down by the indentation stress field (yellow arrow in the magnifited inset image at the indentation depth of *h* = 7.4 nm), which forms a PDL and then retracts back. As the contact area is extended during subsequent loading after the first PDL emission, new shear loops with larger diameters than the previous one (white arrow, *h* = 13.0 nm) evolve, but their nucleation requires the accumulation of more elastic energy, delaying the formation of subsequent PDLs. In the case of a larger initial contact area, the situation is different, as shown for a 200 nm thick Au nanowire in Fig. 3b (see also Supplementary Movie 4). Rather than a PDL being emitted almost immediately, for the larger initial contact, several embryonic dislocations nucleate from the indented region, accompanied by a sudden change of the strain contrast. The dislocation nucleation occurs sequentially along the parallel (*a*) and (*b*) as well as the inclined (*c*) and (*d*) slip planes. The dislocation activities along these two types of slip planes are geometrically necessary to accommodate the shape change made by the indenter and facilitate the associated material transport. The dislocations nucleated along the parallel (*a*) and (*b*) are stored in the plastic zone and one of them is triggered to evolve into a stable shear loop that does not spontaneously shrink back. Similar to a Frank-Read source, the shear loop bulges out in response to the indentation stress field and acts as a source of PDLs. On the other hand, the embryonic dislocations nucleated along the inclined (*c*) and (*d*) slip planes take the form of a half prismatic dislocation loop and glide out of the nanowire (black arrows in Fig. 3b). In bulk materials, it has been shown by MD simulation that this kind of in-plane half prismatic dislocation loop with pure edge character running parallel to the surface can only be annihilated by the pipe diffusion of vacancies from the surface^39^.

As opposed to the stable shear loops, which can eventually punch out a PDL, the remaining short dislocations in the plastic zone are referred to as embryonic dislocations (unless otherwise noted, shear loop refers to the stable one). The observed behaviors indicate the existence of a critical size of the shear loop, above which it becomes stable to grow further. One can notice in Fig. 3a that this critical size of the shear loop scales with the contact area, requiring the accumulation of larger elastic energy to overcome a larger energy barrier as the contact area is extended.

The shear loop that manages to emerge from the highly stressed zone and subsequently acts as a source of PDL distinguishes itself from the other embryonic dislocations in the stressed zone. It has overcome an activation energy barrier arising mainly from two competing energy contributions to the dislocation formation process—the energy cost associated with the dislocation self-energy, which varies with the dislocation line length and curvature (both proportional to the loop size, *d*), and the energy gain due to the work done by the applied load, which varies with the magnitude of the resolved shear stress and the area swept by the dislocation (proportional to *d*^2^)^40,41^ (as detailed in Supplementary Note 1 and Supplementary Fig. 8). In the case of a small contact area, this energy barrier tends to decrease while the nucleation stress increases. This is because the nucleation stress must be high enough to render the work done by a small half-loop larger than the dislocation self-energy. At such high stresses, the energy barrier is small, which results in the formation of shear loops that expand immediately without much activation processes (Fig. 3a). On the other hand, for typical contact radii of a few 10 nm or even larger in most nanoindentation experiments^16,18^, the energy barrier is significantly large. Thus, there is an activation process for the embryonic dislocations attempting to overcome the energy barrier at reduced nucleation stresses. This energy consideration explains the observation that smaller PDLs are emitted quickly, while larger PDLs require prior dislocation activities involving the nucleation of embryonic dislocations and the growth of shear loop(s) out of the highly stressed zone during loading.

### Formation of PDL from shear loops

After its formation, a shear loop can expand on the slip plane where it lies in response to the indentation stress field. However, the expansion is limited to only a short distance as the indentation stress field decreases rapidly with distance (Supplementary Fig. 5). It is hard to tell by TEM observation whether the shear loop emerges on single or multiple slip planes at the beginning. However, the formation of PDLs definitely requires the extension of the shear loop from its original slip plane(s) to all the other {111} slip planes of a glide prism. According to fundamental dislocation mechanisms^36,37^, during the expansion of the shear loop, the glide of edge segments drags the pinned arms to screw orientation, which creates a highly probable situation for the dislocation to cross slip to another highly stressed slip plane. While globally all the {111} planes are equally stressed by the indentation stress field, there might be local and temporal variations in the resolved shear stress on each {111} plane associated with the presence and glide of shear loops. Thus, a shear loop can cross slip, even successively to wrap itself around a glide prism. Finally, the screw segments attract and annihilate each other as they have opposing line directions, resulting in the pinch-off of a PDL. Even though we can detect the cross slip process clearly only for a large shear loop by in-situ TEM observation, the cross slip of shear loop has been regarded as one most plausible mechanism leading to the formation of PDL and shown to be a key process in many MD and dislocation dynamics simulations^25,30^.

The present in-situ TEM observations show that the formation of PDL also involves multiple shear loops forming separately on (*a*) and (*b*) planes rather than the cross slip of a single shear loop. Each shear loop expands on its respective slip plane and reacts with others, resulting in the formation of a large shear loop lying on the two intersecting slip planes. For example, in Fig. 3b, followed by the formation of the first shear loop on (*a*) plane, a second shear loop was formed on the neighboring (*b*) plane, which then reacted with each other, resulting in an extended dislocation structure lying on (*a*) and (*b*) planes. As the indenter is displaced further, the complicated line shape of the extended dislocations evolves, indicating that the pinching-off of a PDL is not facilitated simply by a cross slip of the screw components. Instead, another dislocation emerges on a parallel (*a*) plane during further loading. Finally, one of the two dislocations cross slips onto the parallel (*b*) plane, reacts, and pinches-off a PDL. While the detached PDL glides away from the indentation stress field, the shear loop shrinks back due to the reduced local stress.

We note that the image stress can affect the cross slip events of the shear loop and its effect becomes significant when it intersects the free surface, as in the void growth by PDL formation^42,43^. The constriction of the dislocation line by the image stress at the free surface can form screw segments ending at the free surface. This image stress tends to promote the cross slip of screw segments, causing the sequence of cross slip events to deviate from that described by the Ashby and Johnson model^40^, leading to the formation of smaller PDLs by premature cross slip. However, in nanoindentation, shear loops do not directly intersect the free surface but mostly the contact interface with indenter or embryonic dislocations. Therefore, the effect of the image stress on the formation of PDLs is much weaker than that of the far-field stress from indentation. The image stress from the sidewall surfaces of a nanowire is also negligible because the PDLs slip in the center of the nanowire, which are several tens of nanometer away from the side surfaces.

### Formation mechanisms of PDL studied by MD simulations

**Fig. 4:**
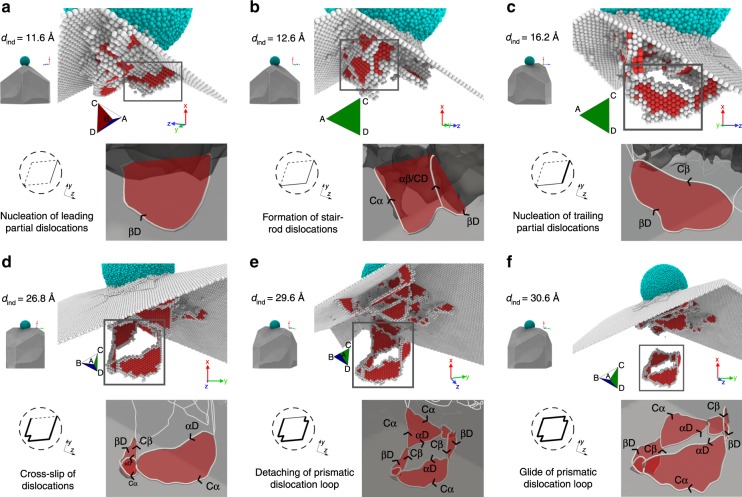
MD simulation showing the atomic-scale mechanism of PDL formation. **a** Nucleation of a leading partial dislocation on an (*a*) plane. **b** Expansion and reaction of the partial dislocation on the (*a*) plane with one formed on the (*b*) plane, leading to the formation of a stair-rod dislocation. **c** Formation of a full shear loop followed by the nucleation of a trailing partial dislocation. **d** Cross slip of the screw segment of the full shear loop from the (*a*) to the (*b*) plane. **e** Reaction with another dislocation segment nucleated on an (*a*) plane. **f** Detachment and glide of a four-sided PDL. The PDL has four additional sides at the acute corners due to the multiple cross slipping of the screw dislocations back and forth, which occurs when the resolved shear stress on the primary and secondary glide planes are nearly identical^43^. Each subfigure consists of four parts. The top row shows the atomistic views. The atoms with face-centered cubic (FCC) stacking are removed for clarity. The cyan atoms represent the amorphous indenter with a radius of 5 nm while the red and gray atoms represent atoms in the hexagonal close-packed (hcp) and undefined structures, respectively. The schematic on the bottom left shows the bottom view of the formation of a PDL. The dashed circle represents the presumed glide cylinder of the PDL, while the dashed lines represent the position of dislocations required to form a PDL. Thin solid lines demarcate partial dislocations, while the thick lines represent full dislocations. The bottom right of each subfigure shows the dislocations (with arrows indicating their line directions) and stacking fault planes, relevant for the formation of the PDL.

With their high spatial and temporal resolution compared to TEM, MD simulations can provide a clearer insight into the underlying atomic-scale mechanism of the PDL formation (Fig. 4, Supplementary Fig. 9 and Movie 5). The MD simulations showed that the formation of PDLs begins with the nucleation of shear loops and continues via two distinct routes, which are: (1) nucleation of multiple shear loops on separate slip planes or (2) cross slip of the screw segments of a shear loop to intersecting {111} slip planes.

On indenting along the nanowire axis with a spherical indenter, dislocation segments with the Burgers vectors on (*a*) or (*b*) planes were nucleated (Fig. 4a) in no preferential order. Figure 4 also schematically shows the bottom view of the formation of a PDL; the dashed lines represent the positions of imaginary dislocation segments forming a closed PDL; the thin-line represents a nucleated partial dislocation segment while a thick line represents a full (although dissociated) dislocation segment. The nucleated partial dislocation segments expanded and reacted (Fig. 4b) to form a stair-rod dislocation according to the reaction: **b**_stair-rod_ = *βD* + *αC* = *αβ*/*CD*, which is mobile only along *CD*.

Such stair-rod reactions are dependent on the angle  between the partial dislocations on the (*a*) and (*b*) plane (the obtuse angle reaction is shown in Fig. 4b; the acute angle reaction results in a stair-rod dislocation with Burgers vector *βα*). As the indenter penetrates further, trailing partial dislocations were nucleated, which, together with the leading partials, constitute shear loops (Fig. 4c). This step is required for the formation of a full dislocation that allows its screw-oriented dislocation segments to cross slip to a different slip plane. The segments of the full shear loop attached to either the surface or the other dislocation segments can attain screw character during further loading. This allows them to cross slip from the (*a*) to the (*b*) plane (as in Fig. 4d), and vice versa. Therefore, the cross slip led to the formation of three sides of a PDL (Fig. 4d). For the formation of the last side of a four-sided rhombus PDL, two possibilities exist; first, another dislocation segment nucleates at the location of the imaginary dislocation site in the schematic (see Fig. 4e), which then reacts with the existing pre-PDL structure, or second, the screw-oriented segments of the existing pre-PDL structure cross slip again. The former occurred in Fig. 4e. Once the four sides of the PDL were formed, it detached from the plastic zone and slipped along the common Burgers vector $CD=a/2[\bar{1}\bar{1}0]$ (Fig. 4f).

One should note that cross slip is also a thermally activated process and its rate is governed by an activation energy. This energy critically depends on the local configuration of the cross slipping segment. For example, the activation energy decreases significantly at a pre-existing constriction such as a jog^44^ or intersections with forest dislocations^45^. Such constriction effects can assist the cross slip of shear loops as their cross slipping screw segments intersect other dislocations in the plastic zone or the contact interface. Previous studies have shown that the activation of cross slip depends more critically on the local stress state^46^ and also the dislocation line length^47^. In general, the critical shear stress for cross slip increases with decreasing line length of the screw dislocation segment^47^. Considering a typical line length of only a few 10 nm for the shear loop appearing at the initial stage of nanoindentation, the critical shear stress required for cross slip becomes comparable to the nucleation stress of the dislocation, which is approximately in the order of 1.5 GPa^47^. Therefore, the cross slip of short screw segments of a shear loop may not be activated easily and competes with another thermally activated process, namely the formation of a new shear loop on an intersecting cross slip plane. Competition between these two thermally activated processes would result in different PDL formation mechanisms as highlighted above.

### Evolution of a PDL array

**Fig. 5:**
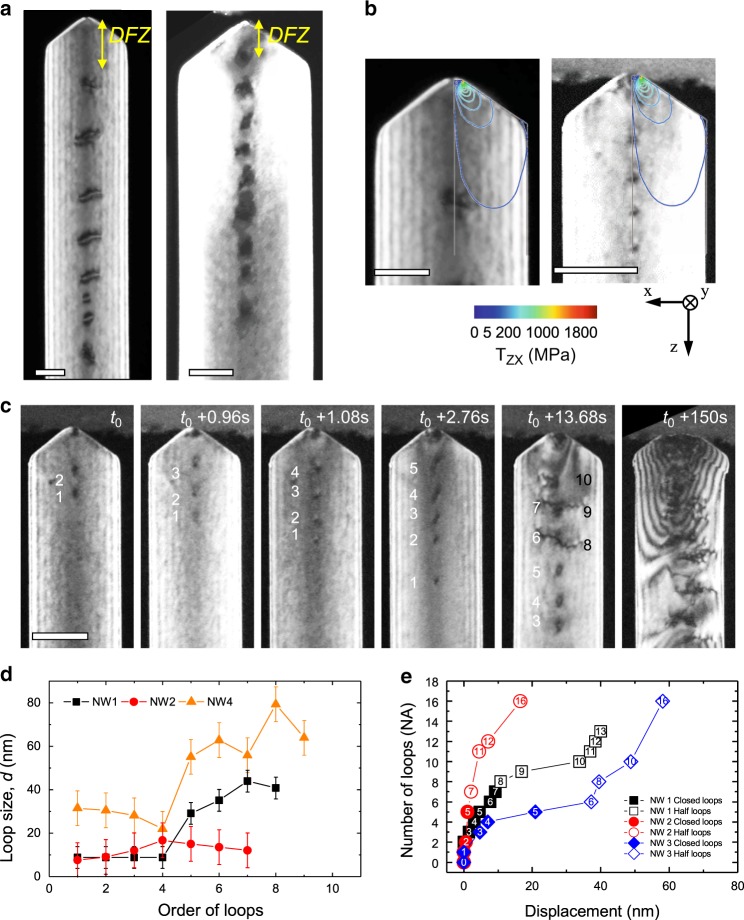
Dynamic evolution of dislocation structure during nanoindentation. **a** TEM DF images representing the typical dislocation structure after indentation. With removal of the indentation stress field by unloading, the stressed zone disappeared and only the coaxial array of PDLs remained. The DFZ is indicated by yellow arrows. **b** Contours of the shear stress (*τ*_zx_) calculated by FEM overlaid on TEM images of indented nanowires with a PDL array. The shear stress at the position of the last PDL is in the order of 1 MPa. **c** Series of TEM DF images showing the transition of PDL from closed (numbered by white labels) to open half-loops (black labels). **d** Change in the size of PDLs numbered in the order of emission. The plot shows a stepwise increase of the loop size. Error bars are spatial resolution limit of diffraction contrast TEM images. **e** PDLs numbered in the order of emission with indenter displacement. After successive emission of around five to seven closed PDLs in series (closed symbols), half loops are emitted after elastic loading (open symbols). Data from three different nanowires (NW1, NW2, and NW3 with diameters of 140, 225, and 425 nm, respectively) are plotted with respect to the indenter displacement. Closed and open symbols represent closed PDLs and open half-loops, respectively. All scale bars are 100 nm.

Our in-situ TEM nanoindentation directly showed the evolution of the dislocation structure during initial plastic deformation, which consists of a highly stressed zone containing embryonic dislocations and shear loop(s) that acts as the source of PDLs, followed by a coaxial array of PDLs that glide away quickly under the influence of the indenter stress field. The characteristic glide behavior of PDLs results in a noticeable feature in the local dislocation structure, a DFZ between the contact area and the last PDL (Figs. 1c and 5a). The size of the DFZ, measured as the distance between the last PDL and the contact, is in the range of 50–250 nm, which is 5–10 times the contact radius in the present experiments.

The stable position of the last PDL in a pile-up array is determined by the balance between the shear stress (*τ*_zx_) of the indentation stress field and the lattice friction stress. Therefore, the lattice friction stress can be estimated by assessing *τ*_zx_ at the position of the last PDL by finite element modeling (FEM), which is measured as around 1 MPa (Fig. 5b) (for details on the FEM results refer to Supplementary Figs. 4 and 5). We observed that as the number of emitted PDLs increases, the size of the DFZ tends to decrease gradually and the last PDL stays closer to the plastic zone because the back stress from the PDL pile-up becomes significant and contributes increasingly to the force balance.

The emission of PDLs proceeds in a discontinuous and burst-like manner. In each burst, a number of the PDLs (around five to seven) are emitted in series (Figs. 5c, d and Supplementary Movie 6). The size of PDLs is measured to remain roughly the same for those emitted in the same burst, and increases in a discrete manner for those emitted in subsequent bursts (Fig. 5d). This suggests that the stored strain energy is first dissipated by the nucleation of a shear loop(s), and the rest or next accumulated energy by its source-like behavior, leading to the multiplication of a series of PDLs at reduced critical stress. This results in a collective and burst-like emission of PDLs until the stored strain energy is exhausted (Fig. 5e). When the dislocation source is shut down, another embryonic dislocation in the plastic zone is triggered to become a new source (shear loop) during subsequent loading, which emits PDLs with increased size (Fig. 5d). This kind of source-controlled deformation behaviour is a signature of small-scale dislocation plasticity as exemplified by the pop-in burst during nanoindentation, but also the discrete strain bursts in sub-micron pillars^48^, and the sudden growth of the plastic zone in front of a crack tip^49^.

The emitted PDLs pile-up in a coaxial array with regular spacing. The inter-loop spacing, especially for those located far from the indenter stress field, is determined primarily by the balance between the repulsive force from the elastic stress fields of neighboring PDLs and the lattice friction stress. When the loop spacings are measured from the indented nanowires and plotted as a function of loop size, it is seen that the loop spacing increases with an increase of loop size (Supplementary Fig. 10). We estimate the lattice friction stress of Au by comparison of the observed inter-loop spacing with the analytical model developed for coaxial and equi-sized circular dislocation loops^50^ (Supplementary Note 2 and Supplementary Fig. 11), which gives 0.2 ± 0.1 MPa, where the error is the standard deviation of the curve fitting. This value is comparable to that assessed from the size of the DFZs and in line with MD simulations^51^. However, we need to consider that in the present experiment the measured lattice friction stress might be overestimated because the PDLs contain stair-rod dislocations, which are dragged by mobile dislocation segments.

Upon unloading, the plastic zone shrinks as most embryonic dislocations and shear loops retract back and disappear (Supplementary Movie 7 and Supplementary Fig. 12). The shear loop (labeled as $D^{\prime}$) in Supplementary Fig. 12 expands during loading but retracts back upon unloading. On the other hand, the PDLs that have traveled beyond the indenter stress field remained unaffected by unloading. This reversible dynamic motion of the dislocations in the plastic zone is responsible for the recovery of plastic deformation^13^ and points out that ex-situ observation of an indented sample may lead to incorrect assessment of the dislocation structure evolving during nanoindentation.

### Transition to helical dislocations

**Fig. 6:**
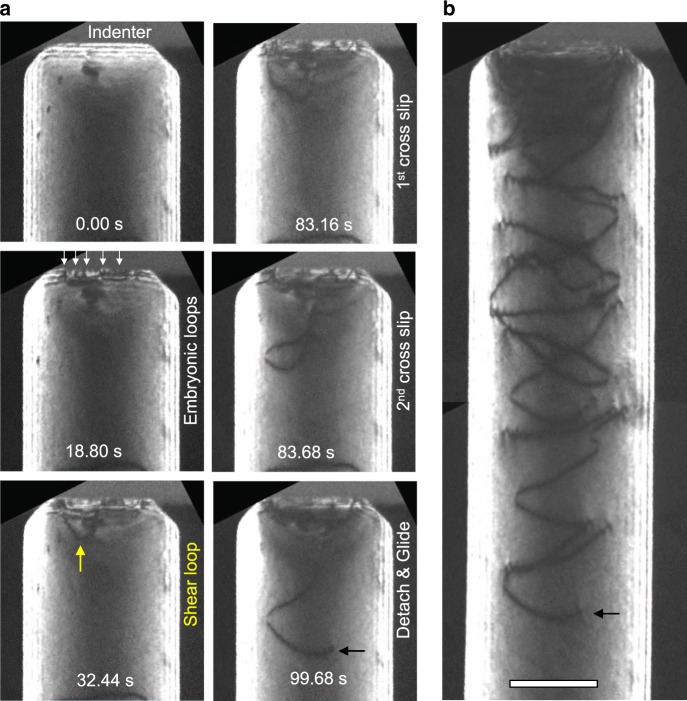
Evolution of a helical dislocation array. **a** TEM DF snapshot images showing the initial stage of helical dislocation formation. In this nanoindentation experiment multiple asperity contacts were formed initially, leading to the nucleation of multiple embryonic loops at different locations (white arrows). Note that for this particular nanoindentation the viewing direction is the [001] direction of the Au nanowire, so that the wedge-shaped tip is seen flat in projection. As the asperity contacts are extended to form a large contact, one of the embryonic dislocations grows as stable shear loop (yellow arrow), dominating the later stage of deformation. The shear loop undergoes multiple cross slips successively, indicating that the cross slip of this large shear loop is energetically favored over the formation of new half-loops. As one of the screw components is detached from the pinning point, it glides out and drags the attached screw component over a large distance. With this dislocation as leading one (black arrow), a series of helical loops is emitted during further loading. **b** TEM DF image showing a helical dislocation array. Easy activation of cross slip of long screw segments enables spiral coiling around the rhombic glide prism before emission from the pinning point. Each helical loop has around two to three pitches of coiling. The TEM movie is provided as Supplementary Movie 8. The scale bar is 100 nm.

Once a larger contact radius (~10–50 nm) is established, either by chance from the beginning or just at a later stage of nanoindentation, the screw segments of a shear loop are extended longer and enable cross slip to another slip plane with further loading. At this step, the competition between the cross slip of the screw segment and the glide of the edge segment determines whether a PDL, a half-loop or a helical dislocation would form: a PDL is formed when the cross slipped segment meets again with the screw dislocation, half-loops form when the two screw components intersect the side surfaces during cross slip, whereas a helical dislocation is formed if the two screw components miss each other (Fig. 6 and Supplementary Movie 8).

A helical dislocation appears as a long spiral, which is a kind of single screw dislocation cross slipped repeatedly onto a set of intersecting slip planes running in parallel. We observed, in most indentation experiments, that helical dislocations are formed preferentially when cross slip is activated at a later stage of indentation with an increase of the contact area. This alternative mechanism for PDL generation, helical coiling of screw dislocations, produces a continuous emission of PDLs without the need for dislocation nucleation at a reduced stress level^43^. Furthermore, they can effectively accommodate the applied strain and the shape change at the top by a cross slip to other slip planes.

### Extension of the plastic zone

The indentation size effect (ISE) described by the Nix-Gao model assumes that all PDLs are contained within a hemisphere with a radius equal to the contact radius *a* (assumed as the plastic zone)^1,27^. The Nix-Gao model predicts the ISE well for large indentations but overestimates the hardness for very small indentation depths. Swadener et al.^52^ suggested that due to the strong repulsive force between GNDs for very small indentations, GNDs would spread beyond the hemisphere assumed; therefore, the model would overestimate the dislocation density and hardness. This so-called spread-out effect has been quantitatively treated by defining an effective radius of *fa*, where *f* is 1 < *f* < 2.4 for several metallic materials resulting in an increase in the storage volume for PDLs^52,53^.

The present in-situ TEM nanoindentation of dislocation-free Au nanowires directly shows that PDLs almost immediately glide out of the indentation stress field and spread far beyond the plastic zone due to the strong repulsive forces between them. As there are no pre-existing, statistically stored dislocations in the Au nanowires, the spreading of PDLs is unimpeded and extends all the way down to the bottom end of the nanowire. This behavior is even more pronounced in FCC crystals such as Au since the lattice friction stress is low. Easy escape of dislocations along inclined (*c*) and (*d*) slip planes to the free surface also increases the spreading distance. The present in-situ observations directly show that in a dislocation-free nanoscale volume the glide of PDLs, although their formation requires a large stress close to the ideal strength, is not impeded and thus does not effectively contribute to work hardening.

### Discussion

The in-situ TEM nanoindentation of dislocation-free Au nanowires revealed that the formation of PDLs proceeds by sequential activation of the nucleation and cross slip of shear loop(s), each of which requires the elastic strain energy to overcome an activation energy barrier. The behavior of the shear loop is in agreement with a pseudo-elastic material response to the indentation stress field before being emitted as a PDL, i.e., bulging out into a half-loop during loading, or shrinking back during unloading. With an increase of the indentation size the critical size and activation barrier for the nucleation of shear loops increases, raising the incubation period of embryonic dislocations in the plastic zone. However, with an increase of the indentation size, the cross slip of a shear loop becomes favorable as its line length increases, leading to a transition in the loop shape from PDL to half-loop and finally helical dislocations in the order of their appearance during nanoindentation. The equilibrium configuration of a coaxial array of PDLs, such as inter-loop spacing and DFZ below the indent, is determined by a balance between the repulsive force from the stress fields and the lattice friction force. As PDLs glide past the indentation stress field and extend the plastic zone due to the strong repulsive force between them, they do not contribute to work hardening as much as in bulk crystals, where their glide is impeded by statistically stored dislocations in the plastic zone. Our direct observations on the formation and glide of PDLs sheds light on the fundamental understanding of the pop-in behavior and related nanoindentation or indentation size effects.

## Methods

### In-situ TEM nanoindentation

The Au [110] nanowires for in-situ TEM nanoindentation experiments were prepared by cutting their supporting SrTiO_3_ (110) substrate into an ~500 μm thick slice using a low-speed diamond saw. In order to prevent contamination of the Au nanowire surfaces during cutting, no lubricating or cooling agent was used. After cutting the substrate, the Au nanowires were observed to stay stable on the substrate without being damaged or contaminated. The slice was subsequently affixed to a half-circle Cu grid using silver epoxy. In order to access the cross-sectional view along the [110] and the [001] directions of the nanowire in TEM, specimens were prepared along these two separate cross- sectional directions. A TEM (JEM-2100F, JEOL Ltd., Tokyo, Japan) operated at 200 kV was used for in-situ TEM experiments. A nanoindentation TEM holder (TEM-nanoindenter, Nanofactory Instrument, Gothenburg, Sweden) equipped with a flat diamond punch tip was used. The indentation tests were carried out using a displacement controlled mode with a displacement rate of 0.5 nm s^−1^. Real-time TEM movies were recorded using a charge coupled device camera (ORIUS 200D, Gatan Inc., Pleasanton, CA) at 25 frames per second.

### MD simulation

The atomistic simulations were performed using the ITAP molecular dynamics (IMD) program^54^. The atomic interactions were modeled using the embedded atom method (EAM) potential for gold developed by Park and Zimmerman^55^. The nanowires were created to match the experimental nanowire shape and aspect ratio. The intersection between the top {111} facets was cut to create a flat surface at the top of the cap in some nanowires. To characterize the size and dimensional effects of the initial plastic deformation of Au nanowires, MD simulation has been carried out with constructing the nanowires with various axial length (*L*_x_), cross-sectional diameter (2 × *a*_NW_) and flat-top half-width (*w*). Motivated by the asperity contact made by the experimental flat punch indenter, spherical indenters with various diameters were used in the present work. The amorphous indenters were made by melting a slab of Au at 1300 K and then quenching it to 300 K with a cooling rate of 10 K ps^−1^. The atomic positions were then fixed and indenters of different radii were cut from the slab. The indenter and the nanowire were then combined to form the simulation setup such that the initial separation between the indenter and sample was the cutoff distance of the potential (5.6 Å) and the indenter was centered on the nanowire axis (Supplementary Fig. 9). The principal axes-*x*, *y*, and *z*-are oriented along [110], [111], and [112], respectively, with the indentation direction along negative *x*-axis. Atoms in the bottom layer of thickness 11.2 Å (=2 × cutoff radius of the potential) were restricted to move only in the plane perpendicular to the indentation axis to ensure that the nanowire is not displaced along the axis of indentation. The initial configurations were then relaxed using two algorithms successively—micro-convergence^56^ for short-range relaxations and fast inertial relaxation engine (FIRE)^57^ for long-range relaxations. The relaxation was carried out until the residual forces per degree of freedom were reduced to a force-norm of 10^−8^ eV Å^−1^. These relaxed configurations were then homogeneously scaled up to the lattice constant at 300 K (*a*_300 K_ = 4.096 Å) and equilibrated for 20 ps using the NVE ensemble. The nanowire was then thermalized at 300 K for 20 ps using the Nosé-Hoover^58–60^ thermostat and barostat.

Nanoindentation was modeled using an NVT molecular dynamics ensemble. A Nosé-Hoover thermostat was used to maintain the temperature at 300 K. The indentation was performed to a maximum indentation depth, *d* = 40 Å, with a constant indenter velocity, *v* = 20 m s^−1^. For a nanowire of length *L*_x_ = 400 Å, *v* = 20 m s^−1^ corresponds to a strain rate of 10^−8 ^s^−1^. The resulting configurations were visualized using OVITO^61^. Common neighbor analysis (CNA)^62,63^ was used to identify local crystallographic defects. The color code in this work assigns green to FCC atoms, red to hcp atoms, and white to other atoms unless specified otherwise. The cyan color is used to identify atoms that belong to the amorphous indenter. In addition, the dislocation extraction algorithm (DXA)^64^ was used to identify dislocations and determine their Burgers vectors.

## Supplementary information

Supplementary Information

Description of Additional Supplementary Files

Supplementary Movie 1

Supplementary Movie 2

Supplementary Movie 3

Supplementary Movie 4

Supplementary Movie 5

Supplementary Movie 6

Supplementary Movie 7

Supplementary Movie 8

## Data Availability

All data generated or analyzed during this study are included in this article (and its supplementary information files), and are available from the corresponding author upon reasonable request.
