# Supplementary material for: In-situ observation of the initiation of plasticity by nucleation of prismatic dislocation loops
Source: Nat Commun. 2020 May 12;11:2367. doi: 10.1038/s41467-020-15775-y (PMC7217955; doi:10.1038/s41467-020-15775-y)
Supplement: Supplementary file 1 — Supplementary Information [file 41467_2020_15775_MOESM1_ESM.pdf]

Supplementary Information

In-situ Observation of the Initiation of Plasticity by Nucleation of Prismatic Dislocation Loops

Lee et al.

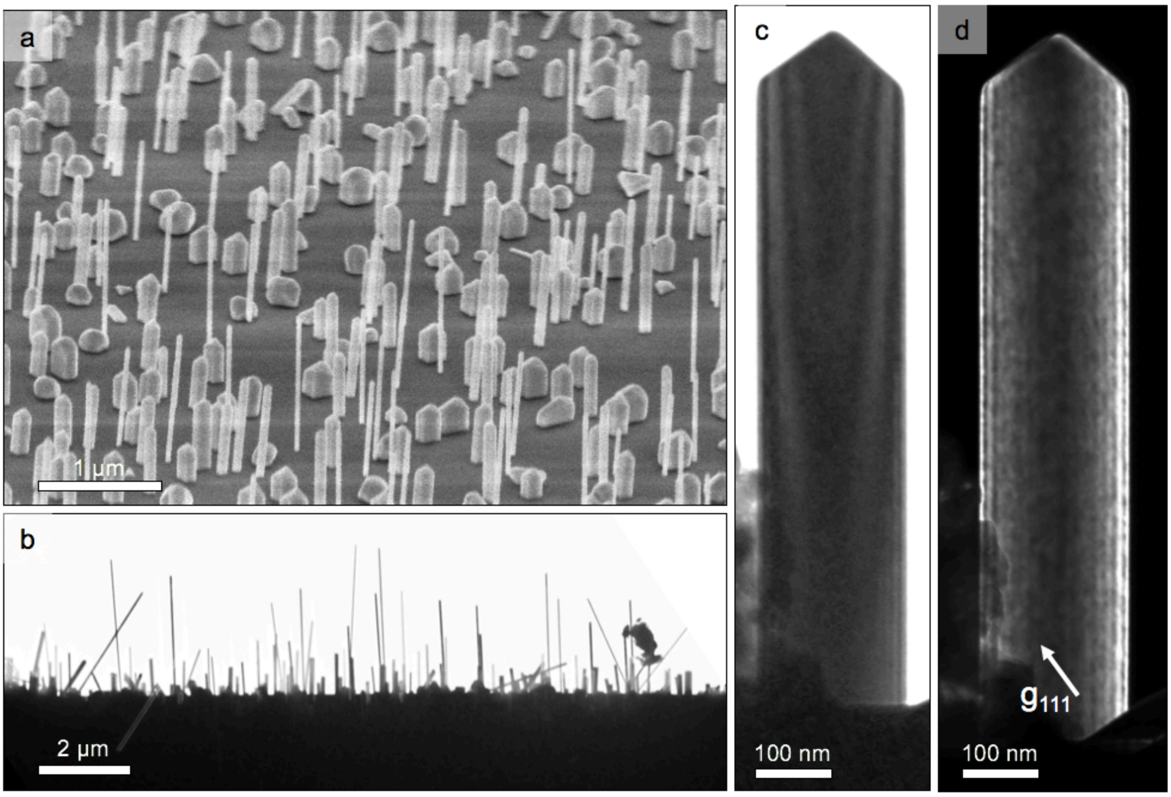

Supplementary Figure 1. [110] oriented single crystal Au nanowires grown on $SrTiO_3$ substrate. a, SEM image of [110] Au nanowires grown on a $SrTiO_3$ (110) substrate. **b,** Low magnification TEM image of a TEM sample. **c,** TEM bright-field and **d,** dark-field image showing that the nanowire is single crystalline and free of dislocations.

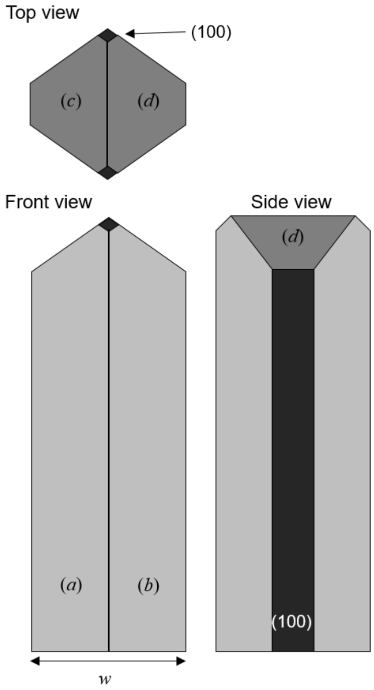

Supplementary Figure 2. Schematic representation of the shape of the [110] Au nanowire. Side surfaces consist of (*a*) and (*b*) slip planes and (100) planes, forming a truncated rhombic cross-section. At the top end of the nanowire two inclined {111} planes, namely (*c*) and (*d*) planes, form a sharp wedge.

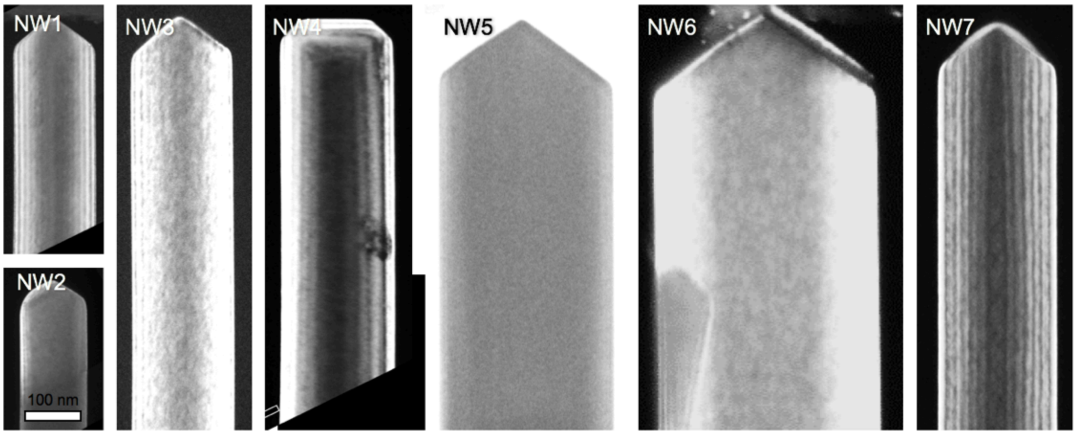

Supplementary Figure S3. TEM dark-field images of Au nanowires used for nanoindentation. All nanowires were perfectly single crystalline and free of dislocations. Only thickness fringes are visible in TEM images. The size of the nanowire, defined as the long diagonal of the truncated rhombus, indicated as *w* in Supplementary Figure 2, varies from 130 nm to 430 nm. The top end of the nanowires was sharp without a flat plateau. In MD simulations, we included a (110) flat plateau with various widths to examine its role in the formation of PDL (unpublished work). The scale bar is 100 nm.

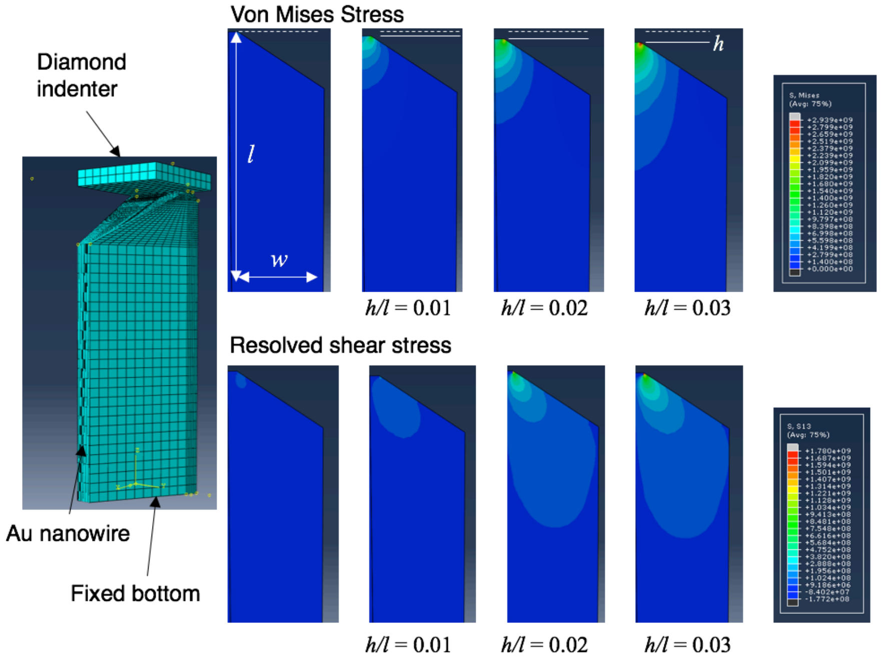

Supplementary Figure 4. Stress distribution in an Au nanowire during nanoindentation calculated by finite element modeling (FEM). The Von Mises stress (upper) and the shear stress (lower) calculated for different indentation depths. The width of the plateau at the top edge is one-tenth of the width of the nanowire.

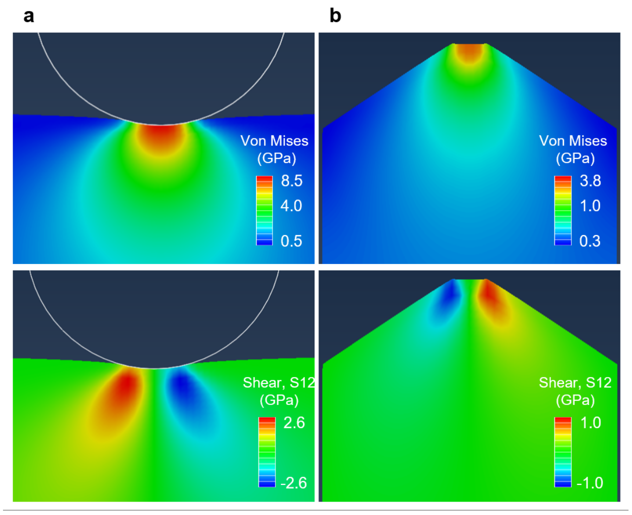

Supplementary Figure 5. FEM calculation of the indentation stress fields for nanoindentation of a semi-infinite flat surface and a wedge-shaped nanowire. a, Flat Au surface indented by a spherical diamond indenter similar to typical nanoindentation. **b,** Wedge shaped Au indented by a flat diamond punch similar to the nanoindentation of an Au nanowire in the present study. For each indentation geometry the Von Mises stress (upper) and the shear stress (lower) are shown. FEM calculation shows that the distribution of the stress fields are similar in both setups although the magnitude of stress in the nanowire is 2-3 times smaller than that near the flat surface.

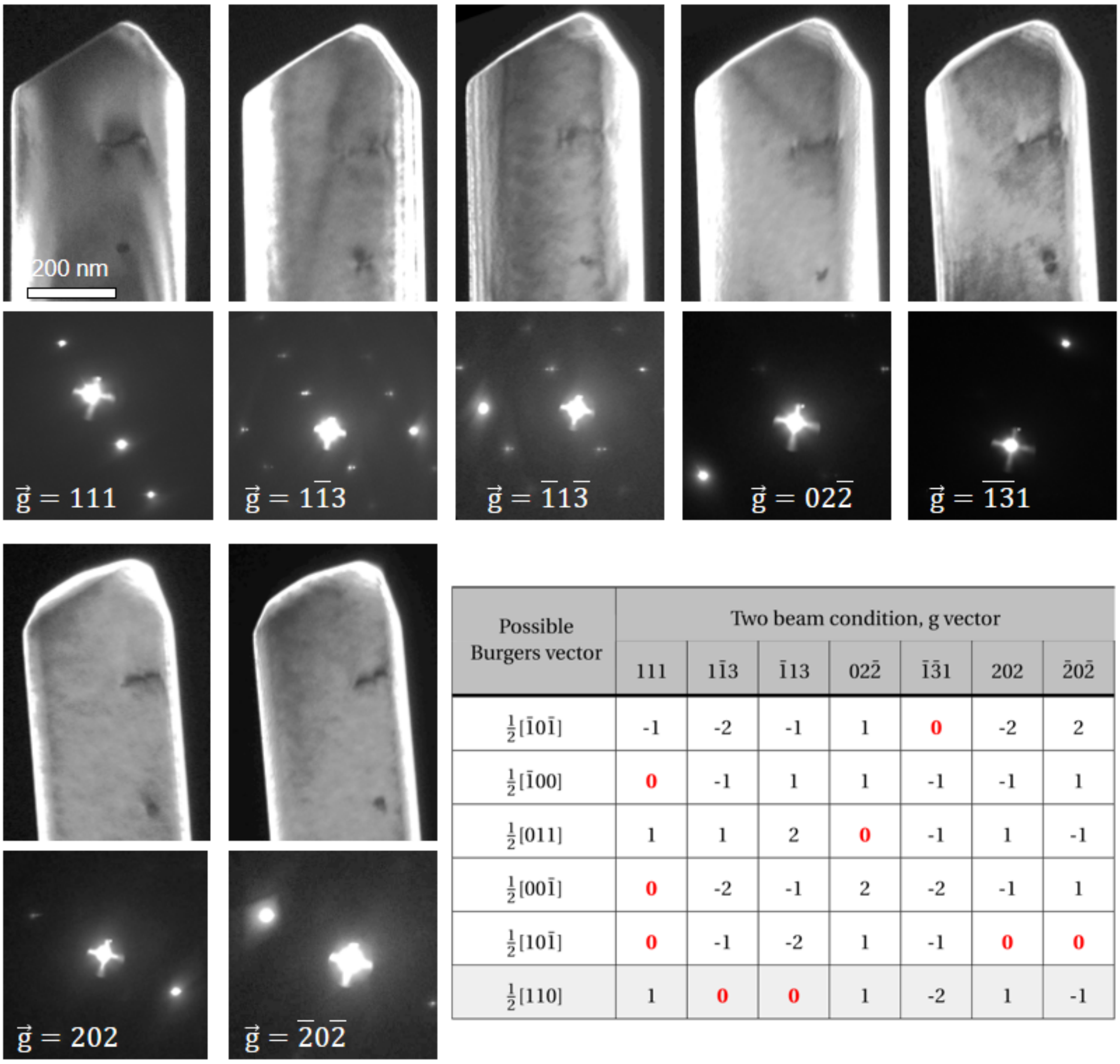

Possible Burgers vector	Two beam condition, g vector						
	111	$1\bar{1}3$	$\bar{1}13$	$02\bar{2}$	$\bar{1}\bar{3}1$	202	$\bar{2}0\bar{2}$
$\frac{1}{2}[\bar{1}0\bar{1}]$	-1	-2	-1	1	**0**	-2	2
$\frac{1}{2}[\bar{1}00]$	**0**	-1	1	1	-1	-1	1
$\frac{1}{2}[011]$	1	1	2	**0**	-1	1	-1
$\frac{1}{2}[00\bar{1}]$	**0**	-2	-1	2	-2	-1	1
$\frac{1}{2}[10\bar{1}]$	**0**	-1	-2	1	-1	**0**	**0**
$\frac{1}{2}[110]$	1	**0**	**0**	1	-2	1	-1

Supplementary Figure 6. The Burgers vector of PDLs determined by applying g·b invisibility criterion. Series of TEM DF images at different two-beam conditions. PDLs are invisible in the second and third images under **g** $= 1\bar{1}3$ and $\bar{1}1\bar{3}$ condition showing that the Burgers vector of the PDLs are $\pm a/2$ $[\bar{1}\bar{1}0]$ which is parallel to the growth direction of the nanowire and the indentation direction. The residual contrast of PDLs in the second and third images is originated from the **g**·**b** × **u** contrast.

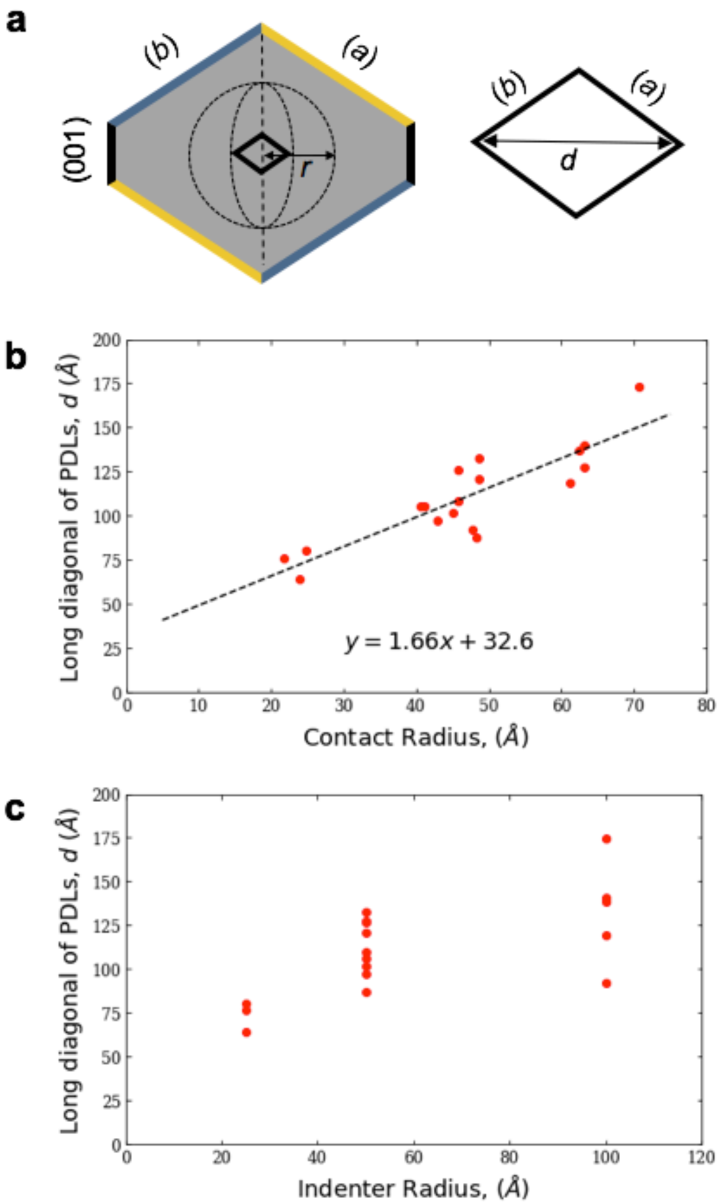

Supplementary Figure 7. Relationship between PDL size and the radius of the contact area. a, Schematics showing an elliptical contact line (outer dotted line) made by a hypothetical round-shaped asperity on the indenter (inner dotted line). A PDL with a rhombic shape is indicated by a thick solid line. The radius of the indenter is defined as r, the long diagonal of PDL as d. **b,** Relationship between the long diagonal of PDLs and the contact radius. A linear fit to the data points is added (dotted line). **c,** The long diagonal of PDLs plotted as a function of the indenter radius (r). The data points on the graphs include data from all different nanowires, indenter types, indenter velocities and indenter radii that were simulated by MD.

Supplementary Note 1. Calculation of the energy change (ΔE) as a PDL bulges out of the contact interface with the indenter

The change in the energy of the system (ΔE) as a dislocation half-loop expands consists of three terms – the energy of a semicircular dislocation loop at the interface with indenter, E^{half}, which is formulated from the self-energy of a dislocation loop in an isotropic, elastic half-space; the additional energy per unit length due to line tension in a curved loop, Γ; the work done by the resolved shear stress component (τ^{RSS}) of the indentation stress field as the dislocation expands, W^{stress}:

$$\Delta E = E^{\mathrm{half}} + \Gamma - W^{\mathrm{stress}}, \tag{1}$$

where

$$E^{\mathrm{half}} = \frac{\mu|\mathbf{b}|^2 R}{8}\left(\frac{2-\nu}{1-\nu}\right)\left[\ln\left(\frac{8mR}{r_0}\right) - 2\right], \tag{2}$$

$$\Gamma = E_{\mathrm{el}}^{\mathrm{mixed}} + \frac{d^2 E_{\mathrm{el}}^{\mathrm{mixed}}}{d\theta^2}, \tag{3}$$

$$\frac{E_{\mathrm{el}}^{\mathrm{mixed}}}{L} = \frac{\mu|\mathbf{b}|^2(1-\nu\cos^2\theta)}{4\pi(1-\nu)}\ln\frac{R}{r_0}, \tag{4}$$

In the equations above, μ is the shear modulus, ν is the Poisson's ratio, m is a geometry dependent correction factor (m = 0.565 for gold), r_0 is the radius of the dislocation core which can be approximated to the magnitude of the Burgers vector **b**, R is the radius of curvature of the dislocation and θ is the angle between the Burgers vector and the line direction. The work done by the applied load can be calculated as:

$$W^{\mathrm{stress}} = \frac{1}{2}\pi R^2 \tau^{\mathrm{RSS}}|\mathbf{b}|, \tag{5}$$

which is the product of the Peach-Koehler force $\boldsymbol{\tau}^{\mathrm{RSS}}|\mathbf{b}|$ and the area the dislocation has swept. Supplementary Figure 7 plots ΔE as a function of loop size for various τ values.

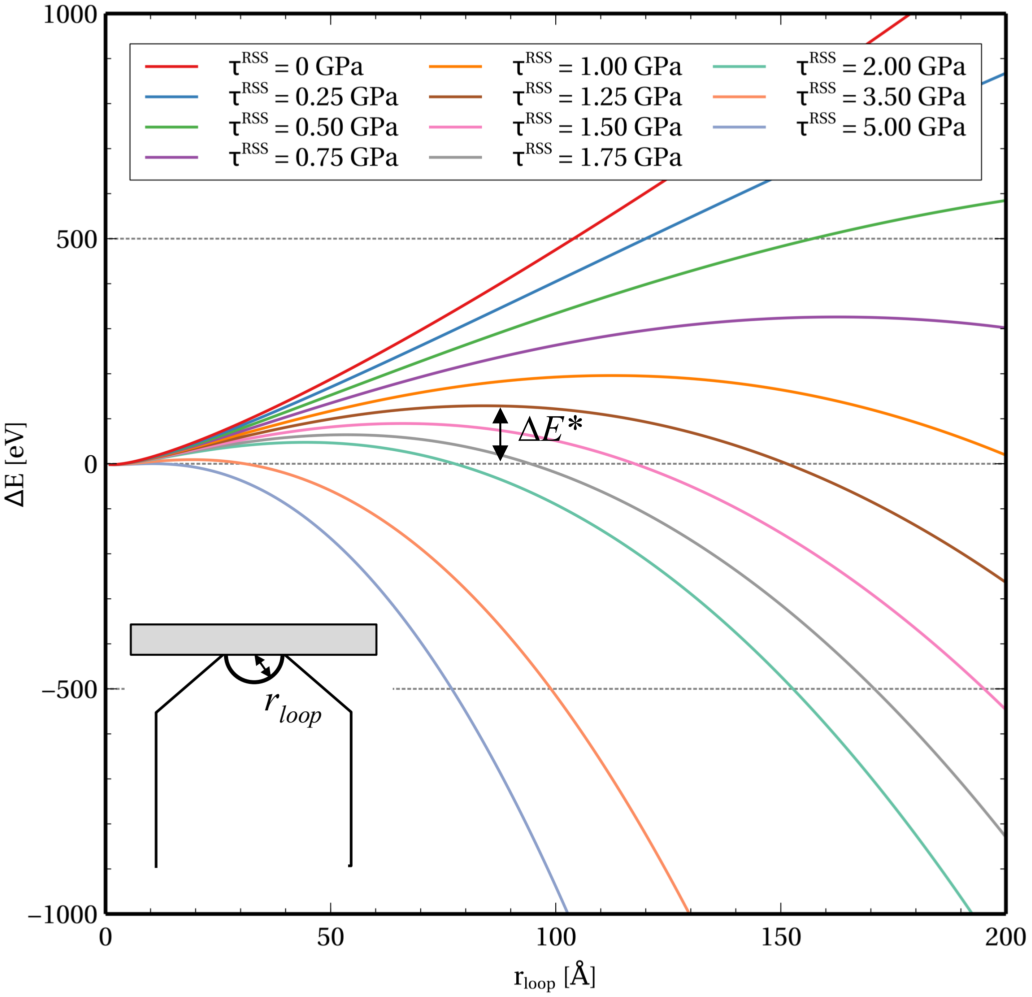

Supplementary Figure 8. The energy change (ΔE) as a dislocation half-loop bulges out of the contact interface with the indenter. Provided the resolved shear stress (τ^{RSS}) is large enough, the formation of an appreciate size of PDL lowers the energy. But an energy barrier ΔE^* must be overcome in order to nucleate the PDL. If the τ^{RSS} increases over 3.5 GPa, the energy barrier ΔE^* vanishes.

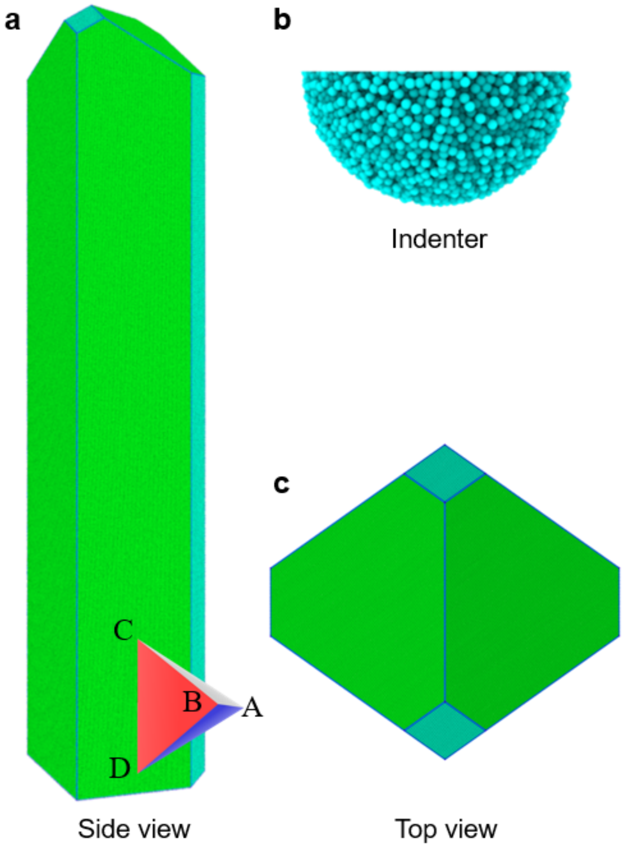

Supplementary Figure 9. Simulation set up for MD simulation. a, Initial simulation setup of the nanowire for the MD simulation. **b,** The rigid indenter used for the simulation. The cyan colored atoms represent immobile atoms with amorphous structure. **c,** Top view images of the nanowires studied in the present work. The top surface consists of two inclined {111} surfaces, (*c*) and (*d*) planes, and {100} surfaces at the corner.

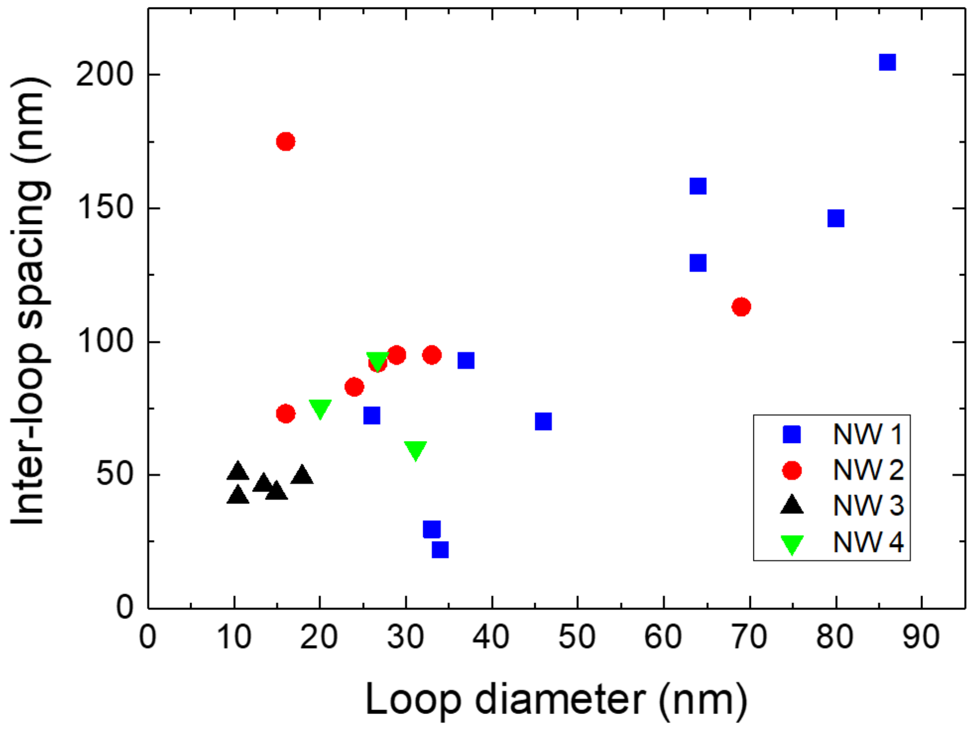

Supplementary Figure 10. Relationship between inter-loop spacing and size of PDLs. The inter-loop spacing of PDLs were measured from TEM images of four Au nanowires after indentation and plotted with respect to the loop size (corresponding to the long diagonal of a rhombic shape loop, *d*). The inter-loop spacing increases as the loop diameter increases, since the elastic strain field becomes stronger at a given distance.

Supplementary Note 2. Determination of the lattice friction stress from the measured inter-loop spacing in an array of PDLs

If there is no external applied shear stress, the force acting on the coaxially aligned array of dislocation loops is only the sum of elastic stress fields from the neighboring dislocations and the lattice friction stress. Xin *et al.* suggested an analytical model that determines the equilibrium spacing within the array of equi-sized circular dislocation loops as a function of the total number and diameter of the loops[1]. For the case of widely-separated dislocation loops, where the loop diameter is smaller than the loop separation, the position of *i*th loops, ζ_i can be written as

$$\zeta_i = \frac{3\pi}{4\bar{d}}\left\{\frac{4}{3}[N^{3/4}-(N+1-i)^{3/4}]+\frac{1}{2}[N^{-1/4}+(N+1-i)^{-1/4}]+\frac{1}{48}[(N+1-i)^{-5/4}-N^{-5/4}]\right\} \quad (6)$$

, where $\bar{d} = 2\pi(1-\nu)\tau_0 d/G|\mathbf{b}|$, d is the diameter of equi-sized loops, G is the shear modulus, $\mathbf{b}$ is the Burgers vector, and N is the total number of dislocations.

We applied this model to our results with the assumptions that: 1) PDLs are equi-sized and circular; 2) the position of the latest PDL (ζ_N) is fixed. From the TEM images showing the array of PDLs, the distance between PDLs (inter-loop spacing) and the distance of the latest PDL from the contact (ζ_N) were measured (Supplemtnary Figure 10a-b). The distances are fitted with Supplementary Equation 6 using least square fitting, which gives $\bar{d}$ for each array of PDLs (Supplementary Figure 10c). We measured $\bar{d}$ from four different PDLs array in different nanowires. With $\bar{d}$ values, the lattice friction stress, τ_0, is calculated as 0.12, 0.29, 0.28, and 0.23 MPa, which gives an average 0.23 ± 0.08 MPa.

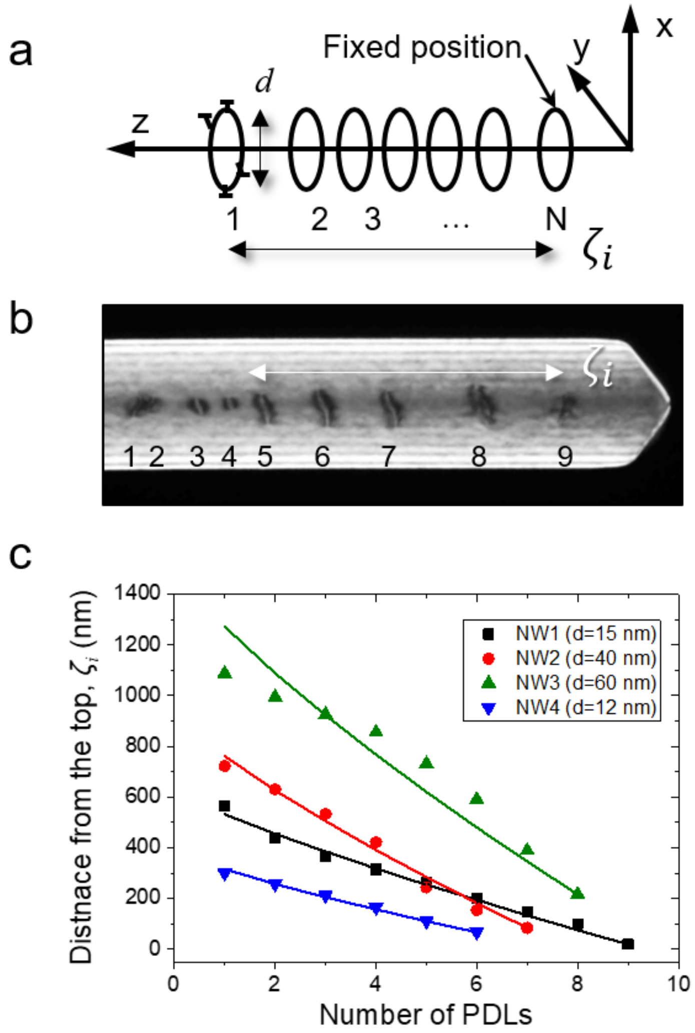

Supplementary Figure 11. Inter-loop spacing of PDLs measured from TEM images and fitting of the data to determine the lattice friction stress. a, Schematic drawing for the equilibrium spacing of a coaxial loop array. **b,** Representative TEM image of a nanowire with an array of PDLs. **c,** Distance from the latest loop for each PDLs. The lines are the least square fitting results of the data points using Supplementary Equation 6.

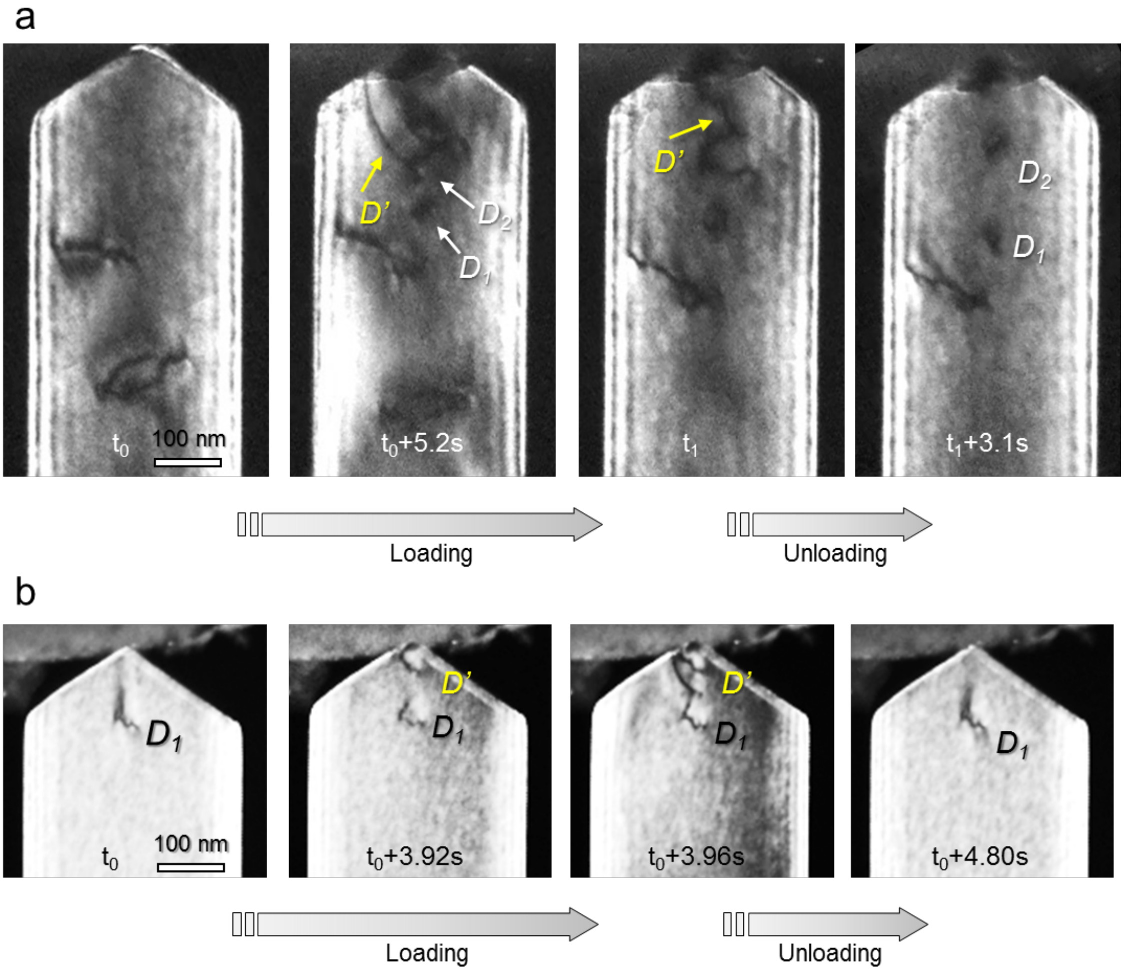

Supplementary Figure 12. Pseudoelastic behavior of dislocations in the stressed zone. a, TEM dark-field images of the indented Au nanowire with two PDLs being stored. Upon reloading of the indented nanowire, two new PDLs D_1 and D_2 were formed and a shear loop, *D*' (indicated by yellow arrow), expands from the plastic zone. With further loading the dislocation half-loop *D*' is dragged by the indentation stress fields. The shear loop *D*' becomes helically coiled after successive cross slip events. Upon unloading the helically coiled dislocation has been completely retracted back and disappeared. **b,** TEM dark-field images showing a similar pseudoelastic behavior of dislocations in the stressed zone (upon unloading the dislocation half-loop *D*' retracts back and disappears).

References

1. Xin, X. J., Daehn, G. S. & Wagoner, R. H. Equilibrium configuration of coaxial prismatic dislocation loops and related size-dependent plasticity. *Acta Mater.* **45**, 1821–1836 (1997).
