# Supplementary material for: In-situ observation of the initiation of plasticity by nucleation of prismatic dislocation loops
Source: Nat Commun. 2020 May 12;11:2367. doi: 10.1038/s41467-020-15775-y (PMC7217955; doi:10.1038/s41467-020-15775-y)
Supplement: Supplementary file 2 — Description of Additional Supplementary Files [file 41467_2020_15775_MOESM2_ESM.docx]

Description of Additional Supplementary Files

**Supplementary Movie 1.** Initial deformation by PDL bursts during pop-in A series of TEM movies showing the initial plastic deformation of single crystal Au nanowires. The nanowires shown in the movie were labeled following Supplementary Figure 3. As the contact is established between the sharp tip of the nanowire and the flat indenter, a series of PDLs is emitted during pop-in and forms coaxial arrays along the loading direction. Followed by PDLs, half-loops and helical dislocations are emitted in subsequent displacement bursts.

**Supplementary Movie 2.** Load-displacement curve obtained by in-situ TEM nanoindentation TEM movie synchronized with load-displacement measurement by in-situ TEM nanoindentation of Au nanowire. After the prolonged elastoplastic loading, two PDLs were formed. The in-situ TEM load-displacement measurement shows that PDL is not the first dislocation initiates the plasticity but one that requires substantial elastoplastic loading for formation after the yield.

**Supplementary Movie 3.** Formation mechanism of PDL under a small asperity contact Formation of the first PDL under a small asperity contact (contact radius ~5 nm). A PDL of ~10 nm in diameter was emitted right after the contact. Before the emission of the PDL a small shear loop was formed readily without activation from embryonic dislocations. The emission of the PDL took less than 0.1 second after contact formation. As the contact radius is extended during subsequent loading, multiple dislocation nucleation events occur before the emission of the next PDLs, resulting in the formation of a zone of high dislocation density. The formation of the second PDL is delayed as the contact area is extended. The movie is played first at 50 frame per second (fps), 5 fps and then 1 fps.

**Supplementary Movie 4.** Formation mechanism of PDL under a large asperity contact A TEM movie showing the formation mechanism of a PDL under a large contact (contact radius > 10 nm). The first critical step is the formation of shear loops on (a) or (b) planes from the embryonic dislocations within the plastic zone. Then, new shear loops are nucleated on other (a) or (b) planes which react with the previous ones. A “lasso”-like shape was formed by cross slip just before the emission of the PDL. Finally, a PDL formed by line closure through the reaction between dislocations.

**Supplementary Movie 5.** MD simulation on the formation mechanism of a PDL MD simulation video showing the formation process of a PDL. The color of atoms is assigned by the common neighboring analysis; fcc atoms are not visible, red to hcp atoms and white to other atoms. Initially, leading partial dislocations with stacking faults are nucleated. Then, the trailing partial dislocations are nucleated releasing dislocations from the contact area. Finally, a PDL is formed through multiple cross-slip and pinching-off of the dislocation.

**Supplementary Movie 6.** Burst-like emission of PDLs A series of TEM movies showing the burst-like emission of PDLs. Several PDLs (less than ten) are multiplied in a single burst by source-like operation of a shear loop. The source operation shuts down after the emission of several PDLs. A new source developed from another shear loop replaces the old one. The PDLs emitted from the newer sources are larger in diameter and show a transition to open half-loop and to helical dislocations.

**Supplementary Movie 7.** Pseudoelastic behaviors of dislocations in the plastic zone A series of TEM videos showing reversible motions of dislocations during cyclic loading, which is responsible for the pseudoelastic response of the indented region. The shear loops under influence of the indentation stress fields exhibit reversible expansion and retraction during loading and unloading, respectively. PDLs which traveled far away from the indentation stress fields are not affected by unloading.

**Supplementary Movie 8.** Formation mechanism of helical dislocation loop A TEM movie showing the formation of helical dislocation loops. The nanowire was viewed along the [001] direction. Initially, a stable shear loop forms and expands with their arms being pinned at the contact interface. Then, the shear loop cross-slips to the other slip planes and forms a long lasso shape. Finally, one of the ends of the shear loop is detached from the contact, resulting the formation of a helical dislocation.
